# Uncommon borderline ovarian tumours: A clinicopathologic study of seventeen patients

**DOI:** 10.4274/jtgga.galenos.2018.2018.0098

**Published:** 2019-11-28

**Authors:** Dilek Yüksel, Caner Çakır, Günsu Kimyon Cömert, Çiğdem Kılıç, Yasin Durmuş, Nurettin Boran, Gökhan Boyraz, Alper Karalök, Taner Turan

**Affiliations:** 1 Department of Gynecologic Oncology Surgery, University of Health Sciences, Etlik Zübeyde Hanım Women Disease Training and Research Hospital, Ankara, Turkey

**Keywords:** Borderline ovarian tumors, survival, stage

## Abstract

**Objective:**

To evaluate uncommon types of borderline ovarian tumors (BOT) and define the clinical, surgical, and pathologic features.

**Material and Methods:**

Seventeen patients who were treated in our hospital between 1990 and 2017 were identified. Patients’ data were collected from the gynecologic oncology clinic electronic database, patients’ files, and pathology reports. Conservative surgery was defined as preservation of the uterus and at least part of one ovary.

**Results:**

The mean age was 47 (range, 22-70) years. Based on histopathologic tumor type, there was mixed tumor in five (29.4%) patients, endometrioid-type in nine (52.9%), seromusinous-type in two (11.8%), and Brenner-type in one (5.9%). Conservative surgery was performed in 4 patients. Two patients with endometrioid BOT had synchronous endometrial pathology, including one (11%) patient with endometrial cancer, one (11%) with endometrial hyperplasia without atypia, and 3 (33%) patients had endometriosis. The median follow-up was 19 (range, 1-137) months. No recurrence was observed during the follow-up period.

**Conclusion:**

In our small volume case series, it could be said that non-serous/non-mucinous BOT has excellent prognosis. However, endometrial pathology should be checked in endometrioid type.

## Introduction

Borderline ovarian tumors (BOT) account for 14-15% of all ovarian neoplasms (1). These tumors usually occur at an early stage, with 25% of cases diagnosed at stages 2-4 according to the staging system defined by International Federation of Gynecology and Obstetrics (FIGO). Considering all BOT cases, the overall 10-year survival rate was reported as 97%. BOTs are tumors of epithelial origin characterized by increased cellular proliferation and mild nuclear atypia; however, stromal invasion is not typical for these types of tumors (2). This group of tumors was described by Taylor in 1929 as “semi-malignant tumors” presenting with peritoneal involvement (3). In FIGO 1971, these tumors were named “low malignant potential tumours” thus separating this entity from ovarian carcinomas (4). They were defined as “atypical proliferative tumors” instead of “borderline tumors” in the 2014 World Health Organization (WHO) classification (5). Based on the epithelial cell type, six histologic subtypes of these tumors have been defined. Serous (50%) and mucinous (45%) tumors are the most common subtypes, whereas endometrioid, clear cell, seromucinous, and Brenner subtypes are seen more rarely (6).

In this study, pure serous and pure mucinous subtypes of BOT were excluded, thereby aiming to define the clinical, surgical, and pathologic features of rarer BOT subtypes, including the endometrioid, seromucinous, and Brenner tumours, and the mixed types.

## Material and Methods

A total of two hundred ninety-three eligible patients, treated between 1990 and 2017, were reviewed as to whether they qualified for inclusion in this retrospective study. The inclusion criteria were a history of surgery and confirmed pathologic diagnosis of BOT, and having had a follow-up examination at the local gynecologic oncology center. Among these, 17 (5.8%) patients with a final pathologic diagnosis of either endometrioid, seromucinous, or Brenner tumors, or a mixed epithelial borderline tumor defined as containing more than one of these five major types of cells including the serous, mucinous, endometrioid, clear cell, and Brenner cell types (7). The patient data were obtained from the computerized database of the gynecologic oncology department and from the pathology reports and patient files, including medical records.

The staging surgery was defined as “abdominal cytology ± infracolic omentectomy ± appendectomy ± peritoneal biopsy” performed at the right and left paracolic sites. Lymphadenectomy was included in the surgical procedure to include the pelvic ± paraaortic regions as per the surgeon's discretion. For women in the reproductive period desiring fertility, a fertility-sparing approach was preferred. The fertility-sparing approach was defined as the preservation of the uterus and a portion of at least one ovary. According to this definition, conservative surgery included either of a unilateral oophorectomy (UO); a unilateral oophorectomy and contralateral cystectomy [UO+contralateral cystectomy (CC)]; a unilateral cystectomy, or bilateral cystectomy. Definitive surgery was accepted to describe procedures that spared neither the uterus nor the ovaries. Staging was performed according to the FIGO 2014 system. In patients who had not undergone a standard staging surgery, the stage of the disease was determined according to intraoperative findings and pathology results. The patients were seen at the postoperative follow-up visits, which were scheduled every 3 months in the first 2 years, then every 6 months until the 5^th^ year, and thereafter once a year. Gynecologic examination, measurement of Ca-125 levels, and whole abdominal ultrasonography were routinely performed during each follow-up visit.

The study was conducted in compliance with the principles and procedures for clinical studies after approval from the Local Ethics Committee.

## Results

The median age of the 17 patients included in the study was 47 (range, 22-70) years. Histopathologic diagnosis was mixed tumor in five (29.4%) patients, endometrioid subtype in nine (52.9%), seromucinous subtype in two (11.8%), and Brenner tumor in one patient (5.9%). The main symptom of the patients was abdominal pain; six (35.3%) patients were referred to the clinic due to this symptom. In addition, four (23.5%) patients had menstrual irregularities and two (11.8%) had abdominal distention. It was found that three (17.6%) patients with no active symptoms were diagnosed during routine examinations. The median pre-operative Ca-125 level was 77 (range, 12-589) IU/mL. The median tumor size was 100 (range, 50-250) mm. The tumor was unilateral in 14 (82.3%) patients. The clinical and pathologic features of the patients are summarized in Table 1.

It was observed that standard staging surgery was performed in 12 patients. Of these patients, four were at stage 1A, one was at stage 1B, and seven were at stage 1C. Stages of the patients in whom staging surgery was not performed were stage IB in one patient and stage 1A in four patients. In three patients with bilateral BOTs, the tumor subtypes were seromucinous in one patient, endometrioid in one patient, and mixed in one patient (patients #4, #11, and #17). No invasive implantations of the tumor or extraovarian diseases were detected. Lymphadenectomy was included in the surgical procedures performed in 11 patients. Both para-aortic and pelvic lymphadenectomies were performed in 10 patients. None of the patients had pathologies in the lymph nodes. The two patients (patient no #1 and #8) who were diagnosed and underwent surgery at an external center were re-staged in our clinic. The pathologic examination results of these patients were reported as mixed tumor and an endometrioid subtype, respectively. The tumors of these two patients were both staged as 1C according to the final pathology examination reports.

Conservative surgery was performed in four patients (patients #5, #6, #7, and #16). Of these patients, three underwent a UO and one underwent a UO+CC. Detailed information about the surgical procedures is presented in Table 2. Sixteen of 17 frozen section examination reports were available, one of which was reported as a malignant epithelial tumor (patient #4) and a discrimination between BOT and malignancy could not be made in two patients (patients #8 and #10). The final reports of the pathologic examination of the biopsy specimens of these three latter patients were reported to be the mixed subtype of BOT in the former patient and endometrioid subtype BOT in the two latter patients, respectively. Two frozen section examination results were evaluated as benign tumors, the remaining 11 (64.7%) were evaluated to be BOTs.

The pregnancy records of four patients who underwent conservative surgeries were not available. Of nine patients who had endometrioid BOTs, three (33%) patients had endometriosis (patients #6, #8, and #13), one (11%) had endometrial carcinoma (patient #9), and one (11%) patient had endometrial hyperplasia (patient #14). No endometriosis or endometrial pathology was identified in the two patients who were diagnosed as having seromucinous tumors. The presence of endometriosis and endometrial pathology in the study patients is summarized in Table 3.

According to the available postoperative follow-up records of the patients, it was determined that adjuvant treatments were not administered to 16 patients. The median duration of follow-up was 19 (range, 1-137) months. Sixteen patients were followed up at least for a period of 9 months. No recurrence was observed in the patients during the follow-up period.

## Discussion

Data on non-serous and non-mucinous BOT subtypes are limited in the literature. These tumor subtypes constitute 3-4% of BOT cases (7). In the 2014 WHO classification, these rare tumor subtypes were histologically classified into endometrioid, clear cell, transitional (Brenner), and seromucinous subtypes (5). Information about the serous and mucinous subtypes is based on a retrospective analysis. In rare borderline tumors, data on the clinical behavior, treatment, and follow-up of the subtypes are very limited and most reports in the literature are case series. BOT is seen more commonly in younger people compared with the occurences of epithelial ovarian cancers. The ages of our patients ranged from 22 to 70 years and their median age was 47 years. Similar age ranges have been reported in other series in the literature (8,9). Infertility is another finding that occurs in 10-35% of BOTs (10). Retrospective data on the infertility history of our study patients were not available. Conservative surgeries were performed in 4 patients in our study. In the literature, the recurrence rates after conservative surgeries are reported at higher rates compared with those rates of the patients undergoing radical surgeries. Morice et al. (11) reported more recurrence in patients treated with ovarian cystectomy compared with those undergoing oophorectomies. In the present study, no recurrence was observed during the median follow-up of 19 months in any of the patients who underwent definitive surgeries or conservative surgeries.

The stage of the disease is the most important factor in determining the prognosis. Unlike ovarian carcinomas, BOTs are detected at earlier stages. In this study, 17 patients with mixed serous or mucinous tumors were evaluated. Similar to the rates observed with pure serous and mucinous subtypes in the literature, all of the 17 patients included in our study were diagnosed at stage 1. In the literature, the 5-year survival rate is reported as 95% in stage 1 tumors; however, there are no specific data on the rare subtypes.

Although 30% of BOTs are asymptomatic, 50-60% of patients present with non-specific symptoms such as abdominal pain or abdominal distention, and 10% of patients have bleeding-associated symptoms (12,13). In our study, eight patients (47.1%) presented with abdominal pain and distention, four patients (23.5%) had menstrual irregularities, and three patients (17.6%) were asymptomatic.

The levels of Ca-125 are elevated in epithelial ovarian cancer and are used in monitoring the patients; however, Ca-125 is not specific for BOTs in terms of diagnosis or follow-up. Ca-125 levels were negative (Ca-125 <35 IU/mL) in 53.8% of patients in a meta-analysis performed by du Bois et al. (12). The median pre-operative Ca-125 level in our study was 77 IU/mL and the available Ca-125 levels of 9 out of 12 patients were over 35 IU/mL. More than half of the patients in our study (53%, n=9/17) had endometriosis and their endometrial pathologies might explain these high levels of Ca-125. Some studies reported that endometriosis and endometrial pathologies could be comorbid with these rare subtypes of BOTs (7). In the present study, the presence of endometriosis was detected in 33% of patients and endometrial pathologies were identified in 22% of patients; however, no endometriosis or endometrial pathologies were present in seromucinosis BOTs, which are known to be associated with endometriosis.

In BOTs, the accuracy of frozen section reports is determined to be between 58-86% and 31% of the cases are diagnosed as benign tumors (14). In a previous study conducted earlier at our clinic, which included all tumor types in the study, reported that the frozen section diagnoses were consistent with 79% of the final pathology reports making a diagnosis of BOT. In that study, the accuracy of the frozen section diagnoses was determined by the tumor type and the tumor diameter. The accuracy rate was reduced if the tumor subtype was mucinous and if the tumor size was over 10 cm (15). Eleven (64.7%) intraoperative frozen section results were reported as BOT. One intraoperative frozen section examination reported a malignant tumor but the final histopathologic diagnosis was BOT.

### Endometrioid borderline tumors

This subtype of tumor, which is defined as an atypically proliferating endometrioid tumor, accounts for 2-3% of BOT cases, being the third most common subtype after the serous and mucinous subtypes. The mean age at diagnosis is 57 years (16,17). The endometrioid subtype was detected in 3% of the patients in our BOT cohort (n=9/293). It is reported in the literature that patients are mostly diagnosed at stage 1, as it was found in our study.

The endometrioid subtype is known to co-exist with endometriosis and endometrial pathologies. The reported rate of co-existing endometriosis was 63% in the study conducted by Hauptmann et al. (2) and 67% in the study by Roth et al. (17). However, Bell and Kurman (16) reported that 36% of patients with endometriosis were diagnosed with an endometrioid BOT subtype. In that study, a complex atypical endometrial hyperplasia was detected in six patients. Uzan et al. (18) reported that the endometrioid subtype of BOT was found to be associated with endometriosis in 19% of patients and with endometrial carcinoma in 6% of patients. Jia et al. (19) identified comorbid endometrial disorders in endometrioid BOT, reporting that endometrial intraepithelial neoplasia was present in five patients (25%), endometrial carcinoma was present in six patients (24%) with endometrial pathologies, and endometriosis was reported in 23% of patients. In our study, we reported endometriosis in 33% of patients, atypical endometrial hyperplasia in 11%, and endometrial cancer in 11% of patients in our endometrioid BOT series. Therefore, endometrial curettage should be recommended if a fertility preserving approach is chosen.

The clinical presentation of the disease and the disease symptoms are not specific, similar to the other subtypes of BOT. Pelvic masses are found in 70% of patients, and abdominal pain and distention is the main symptom in 20% of cases (20). Concomitant endometrial pathologies in the endometrioid subtype of BOT can lead to symptoms such as menorrhagia and menstrual irregularities. In the present study, the main symptom was menstrual irregularities in 33% of patients (n=3/9).

The endometrioid subtype of BOT occurs mostly as a unilateral tumor (16,18,20); however, bilateral tumors account for 3-9% of cases. The bilaterality rate was 11% in our patients (n=1/9). A small number of tumors of the endometrioid subtype of BOT have been reported after conservative surgeries. The standard management of the endometrioid subtype of BOT is bilateral salpingo-oophorectomy with hysterectomy and peritoneal staging surgery. Snyder et al. (21) reported that no recurrence occurred in four patients who underwent conservative surgeries. Uzan et al. (18) performed conservative surgeries in seven patients in their series (5 unilateral salpingo-oophorectomies and 2 unilateral cystectomies) and they reported that tumor recurred twice in one patient who underwent a unilateral salpingo-oophorectomy. In our study, no recurrence was observed during the follow-up period of 80 and 120 months, respectively, in two patients in whom conservative surgeries were performed.

### Borderline Brenner tumors

Brenner tumor or the transitional cell subtype of BOT occurs rarely. It is defined as a transitional-cell tumor composed of urothelial cells arranged in solid cystic groups in the fibrous stroma. Less than 3-5% of these tumors are of borderline or invasive type (5). It is reported in the literature that 95% of Brenner tumors are benign, 3-4% are borderline, and 1% are malignant (22). Borderline Brenner tumors usually originate from benign Brenner tumors and often present with co-existing borderline and benign components. Until 2012, approximately 30 borderline Brenner tumors were reported in the literature (22).

Uzan et al. (22) reported that all 10 patients in their study had unilateral tumors, with the disease being limited to one ovary (stage 1). One of the 5 patients, who was followed-up regularly, was reported to have recurrence and died (22). The Brenner tumor in the patient in our study was a 70-year-old woman with postmenopausal bleeding. An endometrial biopsy revealed a simple atypical hyperplasia after the pathologic examination and her pre-operative Ca-125 level was found as 35 IU/mL. A total abdominal hysterectomy and bilateral salpingo-oophorectomy was performed in this patient who had a mass of 10 cm originating from the left ovary. The frozen section evaluation revealed a BOT; however, without a definition of the tumor subtype. The final pathologic examination reported a borderline Brenner tumor and the stage was determined as IA.

### Seromucinous borderline tumors

The seromucinous subtype of BOT is known as an atypical proliferative serous neoplasm and was previously described as an endocervical type mucinous BOT or as a mullerian mucinous BOT. It is classified as a separate subtype in the 2014 WHO classification (5). It accounts for approximately 5-7% of all BOT subtypes (2). Unlike other endometriosis-related tumors, seromucinous BOT is highy related with benign seromucinous tumours and seromucinous carcinomas. The seromucinous BOT has been suggested to be composed of atypical endometrioid cysts showing mucinous differentiation (23).

The seromucinous subtype of BOT usually arises in young women (age 34-44 years) (24). Bilateral tumors occur in 40% of cases and 20% have peritoneal implantations and lymph node involvements (25). Approximately 30-70% of patients with seromucinous BOT have endometriosis (5).

Prognosis is good even in the presence of extraovarian involvement. There were 2 patients with this subtype in our study and these patients were respectively aged 22 and 45 years. Conservative surgery was performed in the 22-year-old patient and definitive surgery was performed in the 45-year-old patient. Staging surgeries were performed in both patients during the primary surgery. Both patients were determined to be at stage 1C. No adjuvant treatments were given to these patients. There was no evidence of endometriosis-related pathologies or malignancies in either patient. No recurrence occurred during the follow-up periods of 19 and 12 months, respectively. The intra-operative consultation with the pathology department resulted in subtyping the tumor as a mucinous BOT in the young patient and as a serous BOT in the older patient.

### Clear cell borderline tumors

The clear cell BOT subtype accounts for less than 1% of all BOTs and is usually detected in the 59-68 years age range. Most clear cell BOTs are unilateral (5). The disease is usually staged 1 at the time of diagnosis. In this subtype, the prognosis is generally good; therefore, conservative treatment can be offered to selected patients. However, the endometrium should be evaluated with endometrial sampling.

The non-serous/non-mucinous subtypes of BOT present with good prognostic outcomes. However, endometrial pathologies should be screened in the endometrioid subtype. Although the common subtypes of BOT are well-examined, the data are limited for the rare subtypes, usually being reported as case series. Like the frequent borderline ovarian tumours, the uncommon subtypes represent different biologic behavioral patterns and their malignant potential is still uncertain.

## Figures and Tables

**Table 1:**
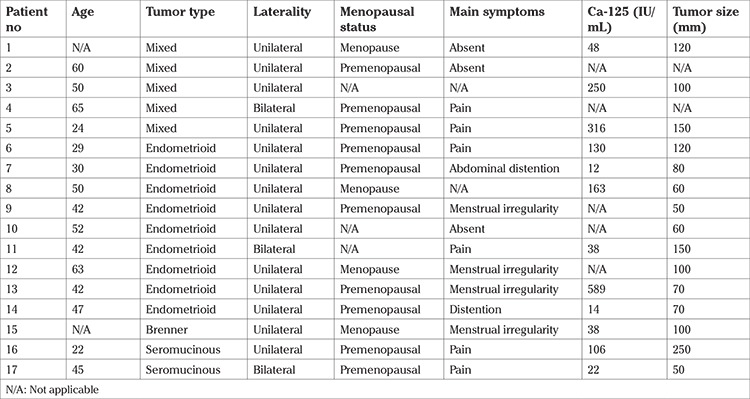
Clinical and pathological features of patients

**Table 2:**
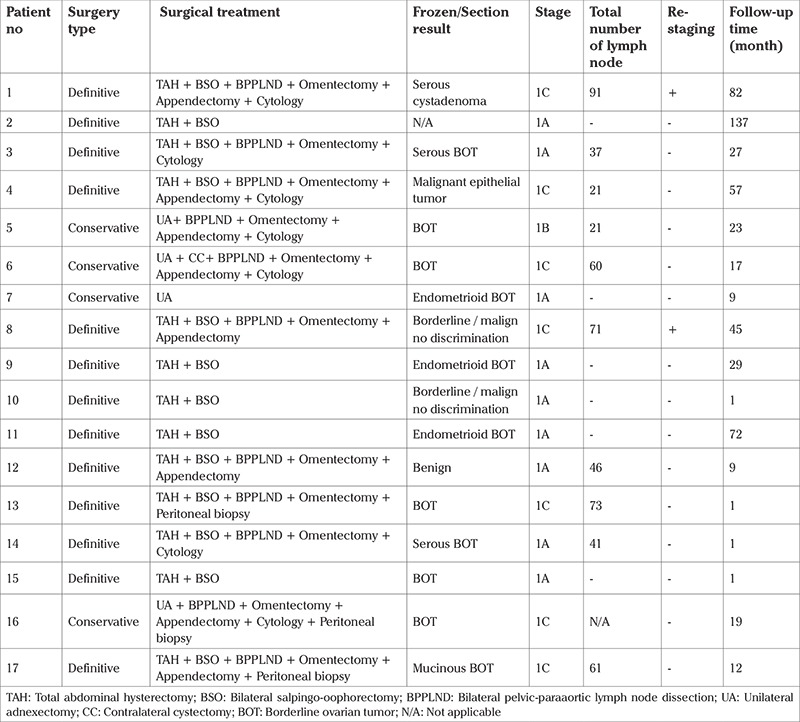
Details of surgical procedure

**Table 3:**
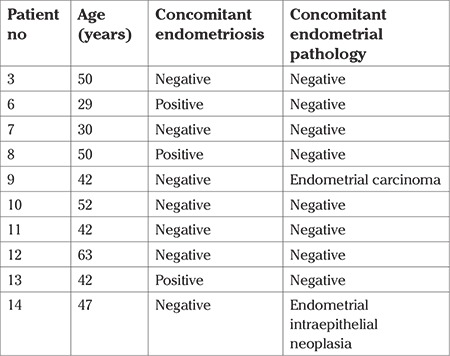
Synchronous endometrial pathologies in endometrioid borderline ovarian tumor
